# Enhancing Role of Nitrogen Fixation in Biogeochemical Cycles of the Pacific Arctic

**DOI:** 10.1111/gcb.70910

**Published:** 2026-05-15

**Authors:** Takuhei Shiozaki, Amane Fujiwara, Eiji Watanabe, Shigeto Nishino, Naomi Harada, Akiko Makabe

**Affiliations:** ^1^ Atmosphere and Ocean Research Institute The University of Tokyo Chiba Japan; ^2^ Research Institute for Global Change Japan Agency for Marine‐Earth Science and Technology Yokosuka Kanagawa Japan; ^3^ Now at Arctic Environmental Research Center National Institute of Polar Research Tachikawa Tokyo Japan; ^4^ Institute for Extra‐Cutting‐Edge Science and Technology Avant‐Garde Research Japan Agency for Marine‐Earth Science and Technology Yokosuka Kanagawa Japan

**Keywords:** Arctic warming, biogeochemical cycles, borealization, nitrogen fixation, sea‐ice loss

## Abstract

The Arctic Ocean is warming rapidly, prompting increased attention to the ecosystem's response to sea‐ice loss and the influx of organisms from lower latitudes. Recent studies have shown that these exogenous organisms are affecting the biodiversity of Arctic ecosystems, but their impact on biogeochemical cycles remains unclear. This study provides evidence that diazotrophs transported from outside the Arctic can significantly contribute to the Arctic new production. From 2015 to 2020, we conducted broad observations in the Chukchi and Beaufort Seas during late summer and autumn, measuring nitrogen fixation rates, diazotroph community composition, and key processes related to new production (nitrate assimilation and nitrification). In 2017, we observed a marked increase in nitrogen fixation, especially in the off‐shelf region undergoing oligotrophication, where it constituted a significant fraction of the new production (interquartile range 10.8%–62.5%, median: 21.5%). UCYN‐A2 (*Candidatus* Atelocyanobacterium thalassa) emerged as the dominant diazotroph across all regions in that year, with a significant positive correlation between its abundance and nitrogen fixation rates, suggesting its key role in the elevated nitrogen fixation. Water mass analyses, sea ice observation, and numerical simulations indicated that UCYN‐A2 likely originated in the Bering Sea and was transported to the Arctic off‐shelf region, and proliferated there with increasing temperature as a result of unusually early sea ice melt in 2017. These findings indicate that as warming and earlier sea ice retreat continue, the influence of exogenous diazotrophs on Arctic biogeochemical cycles can become increasingly pronounced.

## Graphical abstract

Composite mean fields of sea surface temperature and sea‐ice distribution for September during 2015–2024, comparing years with high (2015, 2016, 2021) and low (2017, 2019, 2023) sea‐ice coverage. Earlier sea‐ice melt can promote the advection of diazotrophs (UCYN‐A2 and their haptophyte hosts) from the Bering Sea into the Beaufort Sea basin, where their nitrogen fixation can influence regional biogeochemical cycles (green area).

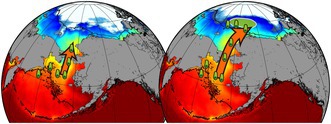

## 1 Introduction

Arctic surface air temperatures are warming at over twice the global average rate (Cohen et al. 2014; Rantanen et al. 2022; Serreze and Francis 2006), with the Arctic Ocean itself experiencing a similar rate of warming in recent years, a phenomenon known as Arctic Ocean Amplification (Shu et al. 2022). This phenomenon is attributed to ice–albedo feedback (ACIA 2004), in conjunction with the increasing inflow of warm water from rivers (Park et al. 2020) and ocean currents from lower latitudes (Shu et al. 2022; Yamagami et al. 2022). The increasing heat content in the Arctic Ocean is potentially driving a substantial reduction in sea ice (Itoh et al. 2026; Muramatsu et al. 2025; Screen and Simmonds 2010; Timmermann et al. 2018), with significant implications for the Arctic Ocean ecosystem. Among the most notable impacts is increased primary production across the region (Ardyna and Arrigo 2020; Lewis et al. 2020), attributed to enhanced underwater light availability and the influx of new nitrogenous nutrients (Lewis et al. 2020). Therefore, understanding the nitrogen dynamics has become essential (Arrigo et al. 2024; Tremblay et al. 2015). Moreover, the Arctic Ocean ecosystem is currently influenced by the migration of subarctic organisms (Alabia et al. 2023; Kortsch et al. 2015; Oziel et al. 2020; Reid et al. 2007; Brandt et al. 2023), a phenomenon associated with increased inflow of water masses from lower latitudes, known as borealization (Polyakov et al. 2020). If these incoming species alter the ecosystem functions, this effect could also impact biogeochemical cycles. Although this possibility has been suggested in some of the previous studies cited above, it has not yet been demonstrated through direct observations.

This study focused on nitrogen fixation in the Arctic Ocean. Nitrogen fixation is a process performed by specific prokaryotes, in which nitrogen gas is converted into ammonia, representing a major nitrogen source in the oceans (reviewed by Zehr and Capone 2020). This process has been studied in tropical and subtropical oligotrophic waters, where it contributes significantly to new production (Capone et al. 2005; Karl et al. 1997; Shiozaki et al. 2017). Recent studies have revealed that nitrogen fixation also occurs in polar regions (Blais et al. 2012; Harding et al. 2018; Shiozaki et al. 2018, 2020; von Friesen, Farnelid, et al. 2025). However, compared with tropical and subtropical oligotrophic waters, where reactive nitrogen is scarce and nitrogen fixation is considered important, the role of nitrogen fixation in the polar biogeochemical cycles has received relatively limited attention, largely because reactive nitrogen is assumed to be more abundant in these regions (Tremblay et al. 2015). In this study, we present evidence that diazotrophs from the Bering Sea actively fix nitrogen and modify the biogeochemical cycle of the western Arctic Ocean. Our results indicate that nitrogen fixation in the Pacific Arctic exhibits considerable interannual variability, corresponding with the distribution of UCYN‐A2 (*Candidatus* Atelocyanobacterium thalassa) and associated symbiotic haptophytes carried by water of Pacific origin. Additionally, we demonstrate that the contribution of nitrogen fixation to new production is markedly higher when nitrogen fixation is enhanced.

## 2 Materials and Methods

Observations and experiments were performed onboard the research vessel (R/V) Mirai in 2015 (MR15‐03, September 6 to October 3), 2016 (MR16‐06, August 30 to September 22), 2017 (MR17‐05C, August 26 to September 21), and 2020 (MR20‐05C, October 8–21) in the Chukchi Sea and the shelf break region of Beaufort Sea. Temperature and salinity profiles were measured using an SBE 911‐plus CTD system (Sea‐Bird Electronics, Bellevue, WA, USA). The salinity sensor was calibrated using bottle data collected throughout the cruise. Water samples were collected in an acid‐cleaned bucket and Niskin‐X bottles. In the euphotic zone, water samples were collected from depths corresponding to 100%, 10%, 1%, and 0.1% of Photosynthetically Active Radiation (PAR), just beneath the surface. The profiles of PAR in the water column were measured using a PRR‐800 (2015 and 2017) or C‐OPS (2016 and 2020) spectroradiometer (Biospherical Instruments, San Diego, CA, USA) immediately prior to water sampling. We also measured sea surface PAR along the cruise tracks using a shipboard quantum sensor (Li‐190R, LI‐COR, Lincoln, NE, USA).

### 2.1 Nutrients and Chlorophyll a

Nitrate, nitrite, ammonium, and phosphate concentrations were determined on board using the QuAAtro 2‐HR system (BL TEC K.K., Tokyo, Japan). Chlorophyll *a* concentrations were also determined on board using a fluorometer (10‐AU, Turner Designs, San Jose, CA, USA) after extraction with *N,N*‐dimethylformamide. The detailed analytical methods of nutrients and chlorophyll *a* are provided in the cruise reports (2015, https://doi.org/10.17596/0001867; 2016, https://doi.org/10.17596/0001870; 2017, https://doi.org/10.17596/0001879; 2020, https://doi.org/10.17596/0002165). The detection limits for nitrate, ammonium, and phosphate were 0.05, 0.07, and 0.003 μM in 2015; 0.04, 0.06, and 0.006 μM in 2016; 0.02, 0.03, and 0.003 μM in 2017; and 0.03, 0.05, and 0.005 μM in 2020. The nitracline depth was defined as the depth at which the nitrate concentration became greater than 1 μM.

### 2.2 Nitrogen Fixation and Primary Production

Nitrogen fixation was determined using the ^15^N_2_ gas dissolution method (Mohr et al. 2010) for all cruises, and primary production was determined using the ^15^N‐^13^C dual inlet technique, following procedures previously reported for this region (Shiozaki et al. 2018). Samples for incubation were collected in duplicate in acid‐cleaned 1.2 or 2.3‐L polycarbonate bottles. In each cruise, ^15^N_2_ gas produced by SI Science (Kanagawa, Japan) was utilized; ^15^N species contamination from the gas has been confirmed to be negligible (Shiozaki et al. 2015). Samples were incubated in an on‐deck incubator filled with flowing surface seawater for 24 h. To calculate quantifiable nitrogen fixation rates, we performed a sensitivity analysis of nitrogen fixation measurements (Gradoville et al. 2017; Montoya et al. 1996). The minimum quantifiable rates in each sample are listed in Dataset S1. Further methodological details are described in the Supporting Information.

### 2.3 Nitrate Assimilation

Nitrate assimilation was determined by the Michaelis–Menten kinetics approach to correct for overestimation caused by excessive use of the ^15^N tracer (Kanda et al. 2003; Shiozaki et al. 2009), with samples collected from the surface and 10% light depth, following procedures previously reported for this region (Shiozaki et al. 2018). Samples for incubation were collected in acid‐cleaned 1.2‐L polycarbonate bottles. Samples were incubated for < 3 h during daytime in an on‐deck incubator. Daily nitrate assimilation rates were calculated from daytime and nighttime ratios of nitrate assimilation determined previously for the Chukchi Sea (Shiozaki et al. 2016). Further methodological details regarding nitrate assimilation are also described in the Supporting Information.

### 2.4 Nitrification

Nitrification rates were also determined using the ^15^N tracer approach (Ward and O'Mullan 2005) as described in our previous study (Shiozaki et al. 2019). Samples for incubation were collected in duplicate in acid‐cleaned 0.3‐L polycarbonate bottles and incubated for 24 h in an on‐deck incubator. The ^15^N/^14^N ratios in nitrate + nitrite of initial and incubated samples were determined using the denitrifier method (Sigman et al. 2001; Kawagucci et al. 2018; Shiozaki et al. 2019). Further methodological details regarding nitrification are also described in the Supporting Information.

### 2.5 DNA Extraction, *nifH* Sequencing, and Quantitative Polymerase Chain Reaction (PCR)

Samples for DNA analysis (2.3 L) were filtered into Sterivex‐GP pressure filter units with a 0.22‐μm pore size (Millipore, Billerica, MA, USA) and stored at −80°C until onshore analysis. Total DNA was extracted using a ChargeSwitch Forensic DNA Purification Kit (Invitrogen, Carlsbad, CA, USA) following the manufacturer's instructions. Partial *nifH* fragments were amplified using a nested PCR approach with samples collected from the surface, where maximum nitrogen fixation typically occurs. The PCR conditions, polymerase, and product purification methods were consistent with those used in previous studies conducted in the same region (Shiozaki et al. 2018).

All samples were sequenced on the MiSeq platform with Reagent Kit v3 (600 cycles) and a Phix control v3 (Illumina, San Diego, CA, USA). Sequenced reads were demultiplexed using MiSeq Reporter v2.6.2 (Illumina). Data analysis was performed with the QIIME2 program v2022.8 (Bolyen et al. 2019), after primer sequences were removed using Cutadapt (Martin 2011). Reads were denoised and clustered based on sequence variants at single‐nucleotide resolution using the DADA2 plug‐in (Callahan et al. 2016). The *nifH* data were translated into amino acid sequences, with non‐*nifH* and frameshifted sequences excluded. The resulting sequences were compared against the *nifH* gene catalog of non‐cyanobacterial diazotrophs (Turk‐Kubo et al. 2023) and the GenBank database using BLASTp.

Quantitative PCR analysis of UCYN‐A2 was applied to all DNA samples and performed using the ABI 7500 Real‐time PCR system (Applied Biosystems, Foster City, CA, USA) for the 2015, 2016, and 2017 cruises, and the StepOnePlus Real‐Time PCR System (Applied Biosystems) for the 2020 cruise. We used previously reported primers and probes for UCYN‐A2 (Thompson et al. 2014). The R^2^ values for the standard curves were 0.994–1.000. The efficiency of the qPCR analyses ranged from 94.2% to 101.8%.

### 2.6 Numerical Experiments

Numerical experiments using a virtual tracer were conducted to visualize Pacific water transport from the Bering Strait using the Center for Climate System Research Ocean Component Model (COCO) v4.9 (Hasumi 2006). The pan‐Arctic regional COCO model used in this analysis has reasonably reproduced major fields of ocean circulation in the western Arctic (Watanabe et al. 2022). The model configuration, experimental design, and corresponding physical hydrographic/circulation results were identical to those reported for previous experiments (Nishino et al. 2023; Watanabe et al. 2022, 2025). The horizontal resolution was approximately 5 km, which is sufficient to represent detailed ocean currents such as the Barrow Canyon throughflow. A virtual passive tracer with a concentration value of 1 (assuming Pacific‐origin water) was continuously released at the Bering Strait from the time of sea‐ice retreat in 2015 (May 22), 2016 (May 16), 2017 (May 13), and 2020 (May 22) (Figure S4). Tracer values throughout the model domain were maintained at zero until these times in each year. The advection and diffusion of the tracer were calculated along with ocean temperature and salinity.

### 2.7 Satellite Data Analyses

Remotely sensed SST data were obtained from the U.S. National Oceanic and Atmospheric Administration (NOAA) Optimum Interpolation SST Version 2 product (Reynolds et al. 2002) (http://www.esrl.noaa.gov/psd/data/gridded/data.noaa.oisst.v2.html). The inter‐annual SST trend was computed by applying a linear regression to each grid. Daily sea‐ice concentration (SIC) data derived from the Advanced Microwave Scanning Radiometer 2 (AMSR2) were obtained from the Japan Aerospace Exploration Agency (JAXA) web portal (https://gportal.jaxa.jp/gpr/). The timing of sea‐ice melt was calculated using daily SIC data for each year. The definition of non‐ice‐covered pixels was defined at SIC < 20%, and the date of sea‐ice retreat was defined as the last date when SIC fell below 20%, prior to the observed annual sea‐ice minimum area. In this study, we used SIC data based on a 5‐day moving average.

### 2.8 Historical Nutrient Data

The R/V Mirai data utilized in this study were acquired in 2002, 2004, 2008, 2009, 2010, and 2012–2020. Additionally, we used data obtained from the R/V Araon cruise during August 2020 as a part of the “Korea–Arctic Ocean Warming and Response of Ecosystem (K‐AWARE)” project. We also used data that have been collected yearly since 2003 by the Canadian Coast Guard Ship Louis S. St‐Laurent under the collaborative framework of the Beaufort Gyre Exploration Project. This study also included data from the Chukchi Borderland Project (Woodgate et al. 2002) conducted by the United States Coast Guard Cutter Polar Star (USA) in 2002 and the International Siberian Shelf Study (Stemiletov and Gustafsson 2009) by using the Yacob Smirniskyi (Russia) in 2008. All data utilized in this study, as well as the observation periods and data sources, are summarized in Table S2.

## 3 Results and Discussion

### 3.1 High Nitrogen Fixation and Its Contribution to New Production During Late Summer

**FIGURE 1:**
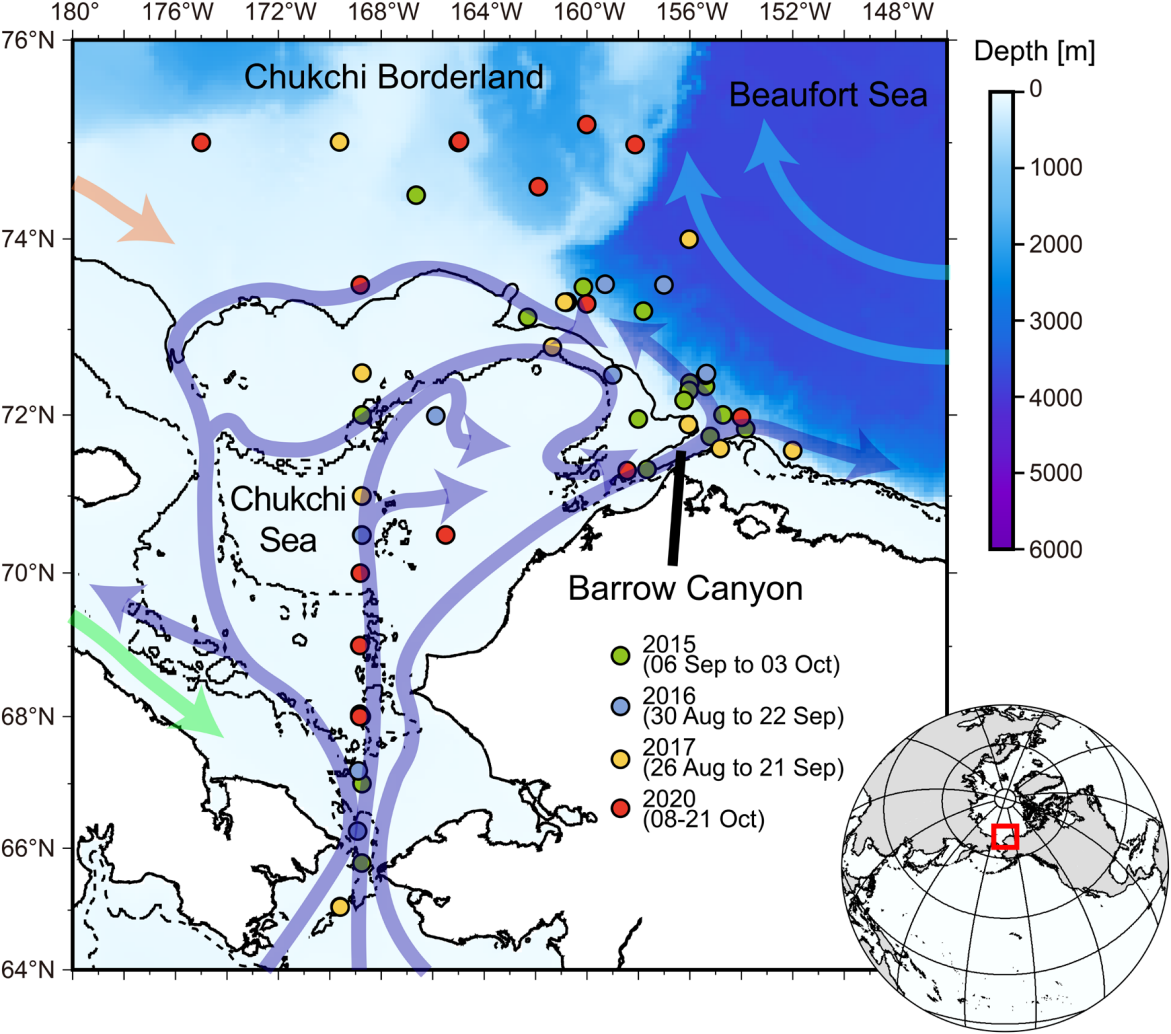
Sampling stations and surface currents in the Chukchi and Beaufort Seas. The current field is based on that of Pickart et al. (2023). Dashed and solid lines indicate 50 and 100‐m isobaths, respectively.

**FIGURE 2:**
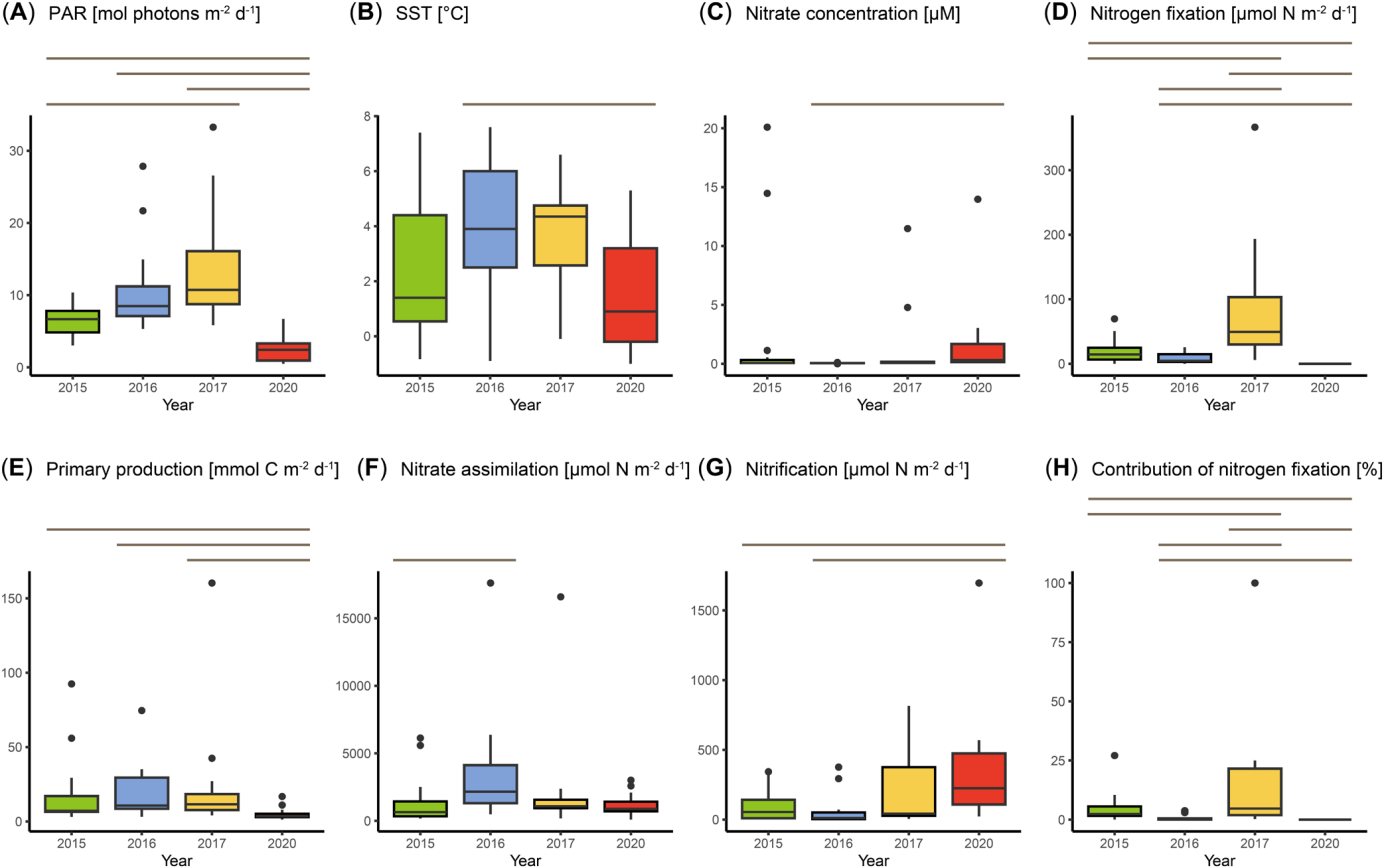
Yearly variations in environmental parameters and biological activity. Box plots show data for (A) photosynthetic active radiation (PAR), (B) sea‐surface temperature (SST), (C) surface nitrate concentration, (D) nitrogen fixation, (E) primary production, (F) nitrate assimilation, (G) nitrification, (H) contribution of nitrogen fixation to new production in each year. Lines on box plots indicate significant differences (*p* < 0.05, Steel–Dwass test). For example, there were significant differences in PAR between 2015 and 2017, 2015 and 2020, 2016 and 2020, and 2017 and 2020.

In this study, we examined nitrogen fixation and primary production in the Chukchi Sea and the shelf break region of Beaufort Sea on four occasions between 2015 and 2020, from late summer to autumn (Figure 1). To elucidate the role of nitrogen fixation in new production, we simultaneously examined the processes related to new production (nitrate assimilation and nitrification rate). According to oceanographic conventions (Dugdale and Goering 1967; Yool et al. 2007), nitrate‐based new production was calculated by subtracting nitrification from the nitrate assimilation rate. The total new production was then estimated by combining the nitrate‐based new production with nitrogen fixation. To our knowledge, this is the first study to investigate the interannual and seasonal variability in new production‐related processes including nitrogen fixation and nitrification rates across a broad area in this region. In 2015, 2016, and 2017, observations were conducted around the same time in late summer, while in 2020, observations took place in autumn. This variation in observation periods led to differences in average PAR values (Figure 2A), with significantly lower PAR in 2020 than in other years (*p* < 0.05, Steel–Dwass test). Sea surface temperatures (SSTs) were also markedly lower in 2020 (Figure 2D). Although average SSTs were similar across the late‐summer cruises in 2015, 2016, and 2017, an expansion of high‐temperature water masses into the off‐shelf region was noted in 2017 (Figure 3A), a year in which the heat content of Pacific‐origin warm water around the Chukchi Borderland reached its highest level in two decades (Muramatsu et al. 2025). During the late‐summer cruises, surface nitrate concentrations were nearly depleted, except at stations in the Bering Strait (Figure S1A–C). In contrast, during the autumn cruise, surface nitrate concentrations increased (Figure 2C), likely due to wind‐induced vertical mixing (Fukai et al. 2025), particularly at stations in the shelf region (Figure S1D).

**FIGURE 3:**
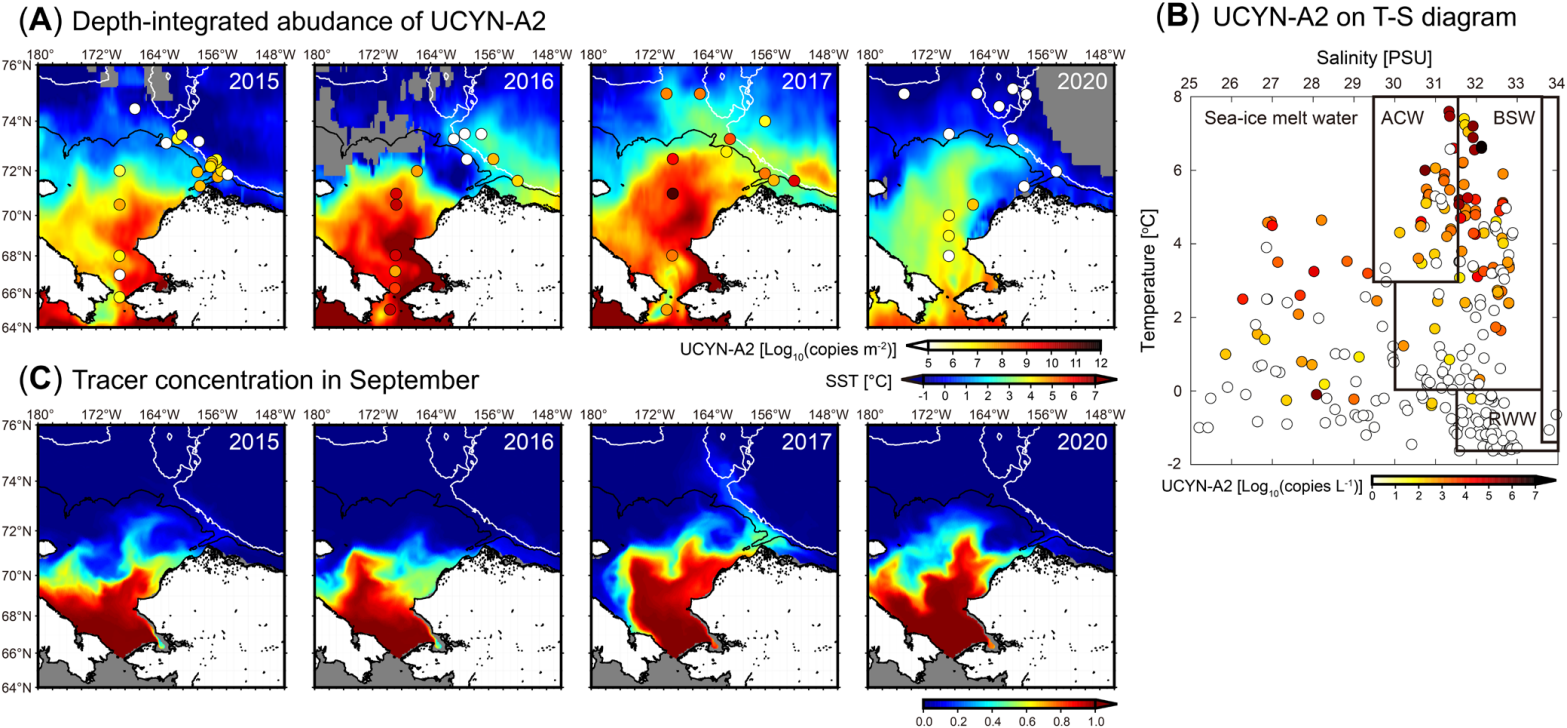
Distribution and potential origin of UCYN‐A2 in the Chukchi and Beaufort Seas. (A) Spatial distribution of depth‐integrated abundance UCYN‐A2 in each year. Background contours indicate satellite‐derived SSTs in September. Gray areas indicate no data. (B) UCYN‐A2 abundance on T‐S diagram. Water classifications were defined by Pickart et al. (2023). ACW, Alaskan Coastal Water; BSW, Bering Summer Water; RWW, Remnant Winter Water. (C) Simulated distribution of a Pacific water tracer released at the Bering Strait from the time of sea‐ice retreat; its concentration in the ocean surface layer in September is shown. Solid black and white lines indicate 100 and 1000‐m isobaths, respectively.

Nitrogen fixation exhibited substantial variation across years and seasons (Figure 2D). During the late‐summer cruises, nitrogen fixation was significantly higher in 2017 than in 2015 and 2016 (*p* < 0.05, Steel–Dwass test), with elevated nitrogen fixation observed not only in the shelf region but also extending to the off‐shelf region (Figure S1). No nitrogen fixation was detected during the autumn cruise. Nitrogen fixation showed no significant correlation with environmental properties such as temperature, salinity, chlorophyll *a* concentration, PAR, or nutrient concentration (Table S1), indicating that in situ environmental factors may not be involved in the interannual variation in nitrogen fixation. In contrast to nitrogen fixation, primary production showed no variation among the late‐summer cruises; however, it was significantly lower in autumn than in the late‐summer cruises (Figure 2E; *p* < 0.05, Steel–Dwass test). Across all years, primary production showed a significant positive correlation with surface chlorophyll *a* concentrations (Table S1, *p* < 0.001). Nitrate assimilation and nitrification rates also showed a little variation among the late‐summer cruises (Figure 2F,G). During these cruises, the nitrate assimilation rate was correlated with surface chlorophyll *a* concentration and depth‐integrated primary production (Table S1, *p* < 0.001). In contrast, no significant correlation was observed during the autumn cruise, implying that nitrate assimilation may primarily be carried out by non‐primary producers (Fouilland et al. 2007), unlike during the late‐summer cruises. Nitrification rates tended to be higher in autumn; thus, as nitrification in this region is inhibited by light (Shiozaki et al. 2019), it was likely enhanced during autumn when PAR levels were lower. We calculated the contribution of nitrogen fixation to new production at each station (= nitrogen fixation/[nitrogen fixation + nitrate assimilation—nitrification]). This contribution was generally higher in 2017 (Figure 2H), a year with notably high nitrogen fixation, particularly in the off‐shelf region (17.0%–100%), with the exception of one station (St. 74; 4.6%). These high values are comparable to those reported in subtropical oligotrophic oceans (Karl et al. 1997; Raimbault and Garcia 2008; Shiozaki et al. 2017), indicating an increased role of nitrogen fixation in the new production of off‐shelf waters in 2017.

### 3.2 Nitrogen Fixation by UCYN‐A2 and Its Source From the Bering Sea

To examine the factors influencing interannual variation in nitrogen fixation, we further analyzed diazotroph communities in the surface water, where nitrogen fixation was widely detected during the late‐summer cruises. Diazotroph community composition varied significantly across the research cruises (Figure S2). In 2015, Amplicon sequence variants (ASVs) homologous to the *nifH* gene of NB3‐Pelobacter (Brown and Jenkins 2014; Turk‐Kubo et al. 2023) were dominant at most stations. In 2016, UCYN‐A2 was prevalent at most stations in the shelf region; however, non‐cyanobacterial diazotrophs, including NB3‐Pelobacter, PM‐RGC_gene_1205376, and AEA49320, were dominant in the off‐shelf region. In 2017, UCYN‐A2 was the dominant diazotroph at most stations, including the off‐shelf region. In 2020, UCYN‐A1 and UCYN‐A2 were the major diazotrophs at the shelf‐region station 7 and the off‐shelf station 10, respectively, whereas non‐cyanobacterial diazotrophs, including NB3‐Pelobacter, PM‐RGC_gene_1205376, and CE2_5m_1_g, were dominant at other stations. These results imply that UCYN‐A2, which was dominant in 2017, may have contributed to the nitrogen fixation anomaly observed that year.

We also quantified the *nifH* gene of UCYN‐A2 and detected it in all cruises (Figure 3A). Notably, UCYN‐A2 was widely found at high abundances in the off‐shelf regions in 2017, whereas in other cruises, it was primarily confined to the shelf region, except around Barrow Canyon. These distributions were consistent with the diazotroph community composition results. A significant positive correlation was found between nitrogen fixation and UCYN‐A2 abundance in 2017 (Figure S3A, *p* < 0.05), implying that nitrogen fixation was primarily performed by UCYN‐A2. As previously reported (Shiozaki et al. 2023), UCYN‐A2 abundance was significantly positively correlated with water temperature (Figure S3B, *p* < 0.05). Therefore, the low abundance of UCYN‐A2 observed in 2020 was likely attributable to the lower water temperatures during that period. Additionally, low light levels in autumn may have contributed to their reduced abundance. UCYN‐A2 has long been known to associate with photosynthetic organisms, and a recent study has shown that it functions as an organelle (Coale et al. 2024), implying that the photosynthetic activity of its host declines under low light conditions. These findings imply that it is very difficult for UCYN‐A2 to survive during winter in the Arctic Ocean.

Next, we investigated the origin of UCYN‐A2 that reached the Arctic Ocean. High UCYN‐A2 abundance was detected in water masses identified as the Alaskan Coastal Water and Bering Summer Water (Pickart et al. 2023), as indicated by a temperature–salinity diagram (Figure 3B). Therefore, UCYN‐A2 likely traveled northward through the Bering Strait into the study region. We further conducted yearly numerical experiments which adopt the Pacific water tracer released at the Bering Strait from the timing of sea‐ice retreat. Notably, sea‐ice retreat in the Chukchi shelf region occurred significantly earlier in 2017 than in other years (Figure S4). By September in 2015 and 2016, when the cruise observations were performed, the Pacific water tracer had barely reached the region north of Barrow Canyon (Figure 3C). However, in 2017, a substantial amount of tracer extended to the Chukchi Borderland region, and its distributions corresponded closely with the observed distribution of UCYN‐A2. Notably, UCYN‐A2 abundance in the Chukchi Borderland region appears to be relatively high compared with the tracer concentration. Because the tracer experiments did not account for particle growth during transport, this mismatch could be explained if UCYN‐A2 increased after entering the Arctic Ocean. Indeed, transport from the Bering Strait to the Chukchi Borderland is estimated to require more than 3 months, making it unlikely that UCYN‐A2 did not proliferate during transit. As implied by the relationship between UCYN‐A2 abundance and water temperature, increasing temperature may have stimulated its growth. Taken together, these results suggest that UCYN‐A2 was transported from the Bering Sea into the Arctic Ocean while undergoing population growth and contributed to nitrogen fixation there.

### 3.3 Enhancing Role of Nitrogen Fixation in New Production due to Climate Change

This study suggested that UCYN‐A2 transported from subarctic regions could contribute to nitrogen fixation and new production, particularly in the off‐shelf region. Note that in 2016, water temperatures were relatively high and UCYN‐A2 abundances were elevated, particularly in the shelf region, where UCYN‐A2 was the dominant diazotroph. Despite this, nitrogen fixation rates in 2016 were lower than those observed in 2017. One possible explanation is inhibition of nitrogen fixation by enhanced nitrate availability associated with vertical mixing on the shelf (reviewed in Knapp 2012). Indeed, nitrate assimilation rates on the shelf in 2016 tended to be higher than those in 2017. Sporadic vertical mixing occurs on the Chukchi shelf region, enhancing primary production (Nishino et al. 2015). Under such conditions, nitrogen fixation may play a relatively minor role in the biogeochemical cycles. In contrast, primary production and nitrate‐based new production (= nitrate assimilation—nitrification) tended to be lower in the off‐shelf region than in the shelf region, except in 2020 (Figure S5A,B), due to the significantly deeper nitracline in the off‐shelf area compared to the shelf (Figure S5C). Nitrogen fixation is therefore likely to be more important in the off‐shelf region where nitrogen supply from deeper waters is limited.

**FIGURE 4:**
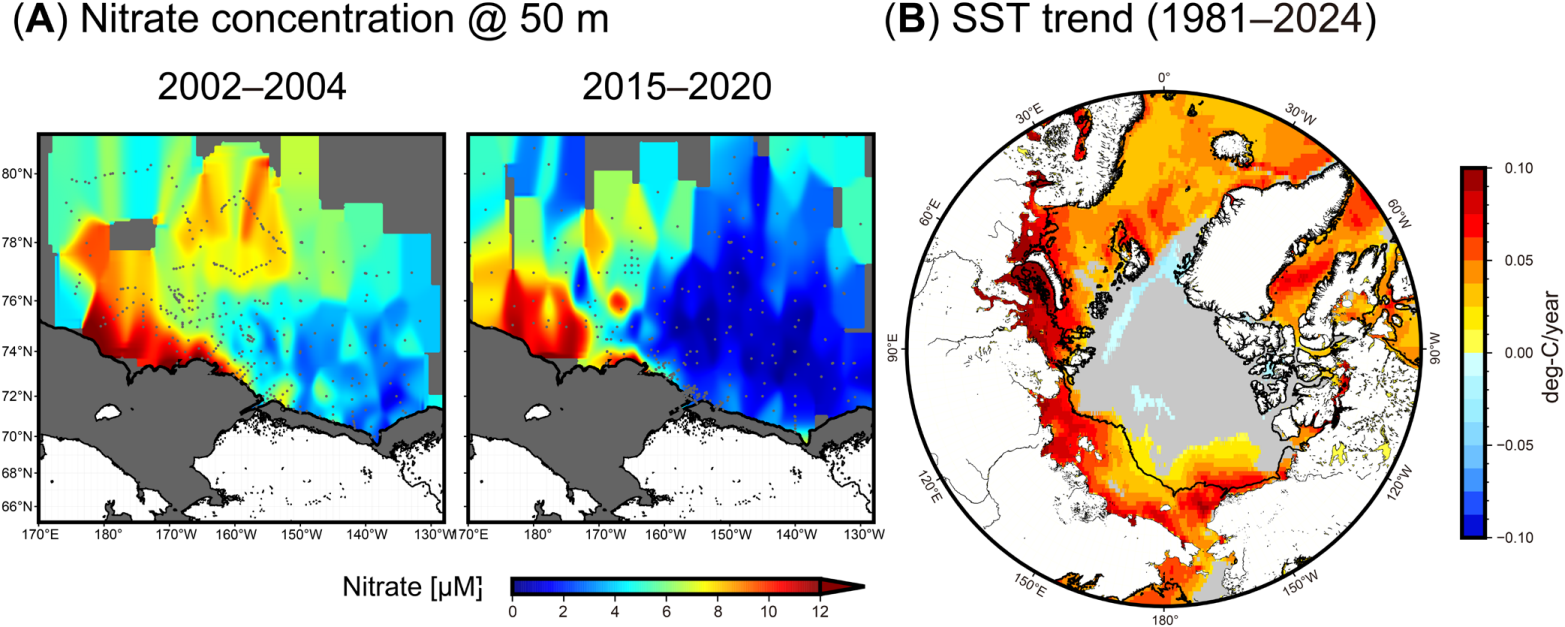
Changes in nutrient fields in the Chukchi and Beaufort Seas and SSTs in the pan‐Arctic Ocean. (A) Nitrate concentration at 50 m during 2002–2004 and 2015–2020. (B) Trends in SST during 1981 and 2024. Only significant trends (*p* < 0.05) are shown here. Solid lines indicate 100‐m isobaths. Dots on the map (A) represent data points.

Previous studies have reported that the basin region of the Beaufort Sea has undergone surface oligotrophication and nitracline deepening, driven by strengthened circulation linked to sea‐ice reduction (McLaughlin and Carmack 2010; Nishino et al. 2013; Polyakov et al. 2020). Those studies analyzed data only up to 2010, with the exception of Polyakov et al. (2020), and did not examine trends over the most recent decade. The present study therefore examined the recent nutrient field up to 2020. Between 2015 and 2020, particularly east of 160° W, nitrate concentrations at a depth of 50 m were found to be extremely low (< 0.5 μM) (Figure 4A). This oligotrophic area has expanded significantly compared to 2002–2004, when the Beaufort Sea retained extensive summer sea ice. Recent findings indicate that the circulation field has shifted eastward due to ongoing sea‐ice reduction (Nishino et al. 2023), yet the trend in oligotrophication in the basin region persists. Pacific‐origin water flowing inward from the Bering Sea reaches the basin area via multiple pathways (Figure 1), but primarily via the Barrow Canyon (Itoh et al. 2013). Given that UCYN‐A2 is likely supplied from the Bering Sea to the Arctic, an earlier sea‐ice melting period, such as that observed in 2017, increases the likelihood of UCYN‐A2 being transported to the basin region. The timing of sea‐ice retreat in the study area has advanced over the past several decades (Markus et al. 2009), likely linked to rising heat transport and content in the downstream regions (Muramatsu et al. 2025; Itoh et al. 2026). Our satellite data analysis shows a significant increase in surface‐water temperatures across the Arctic, including our study area and the oligotrophic Beaufort Sea (Figure 4B). UCYN‐A2 abundance in the Arctic Ocean was significantly positively correlated with temperature, implying that rising water temperatures in the Arctic Ocean promote the growth of host organisms for UCYN‐A2. Correctively, the importance of nitrogen fixation in the Arctic Ocean is increasing compared to the period when perennial sea ice extensively covered the region.

We found that the contribution of nitrogen fixation to new production on the Pacific side of the Arctic Ocean could be increasing due to warming, sea‐ice reduction, and borealization. While warming (Figure 4B) and sea‐ice reduction are widespread phenomena across the Arctic (Ardyna and Arrigo 2020; Cavalieri and Parkinson 2012), environmental conditions on the Atlantic side differ notably from those on the Pacific side. On the Atlantic side, Atlantic water flows directly into the Barents Sea via the Norwegian Sea, where borealization is more pronounced compared to the Pacific side (Ingvaldsen et al. 2021). However, the inflow of Atlantic water and active mixing in the Barents Sea create conditions that facilitate nutrient supply to the surface (Polyakov et al. 2017, 2020). Additionally, circulation patterns on the Atlantic side are less prone to oligotrophication, unlike conditions observed in the Beaufort Sea (Juranek 2022; Polyakov et al. 2020). Therefore, the phenomena described in this study are unlikely to occur on the Atlantic side. Conversely, it appears that the significant contribution of nitrogen fixation to the Arctic ecosystem is predominantly limited to the Pacific side.

The increasing contribution of nitrogen fixation indicates a significant change in the Arctic nitrogen cycle, but its impact extends beyond nitrogen alone. Nitrogen fixation produces reactive nitrogen from nitrogen gas while simultaneously consuming phosphates. Consequently, in subtropical regions where nitrogen fixation is active, previous studies have reported a decline in phosphate concentrations (Moore et al. 2009; Wu et al. 2000). Notably, in this study, the surface phosphate concentrations (average 0.50 ± 0.01 μM) at stations with a high contribution (≥ 17.0% new production) of nitrogen fixation in the off‐shelf region during 2017 were significantly lower compared to the same season in 2015 (0.55 ± 0.05 μM) and 2016 (0.53 ± 0.03 μM) (*p* < 0.05, Wilcoxon rank sum test). This result implies that increased nitrogen fixation alters phosphate concentrations in the Arctic Ocean, thereby influencing the regional phosphorus cycle. Notably, the study area is characterized by active denitrification and a low N/P ratio (Yamamoto‐Kawai et al. 2006); increased nitrogen fixation may therefore modify this N/P balance.

This study highlights the importance of nitrogen fixation by UCYN‐A2 in the western Arctic Ocean. However, the elevated nitrogen fixation rates observed here cannot be attributed exclusively to UCYN‐A2. For instance, high nitrogen fixation rates were also detected at the depth of 0.1% PAR in 2017, where UCYN‐A2 was not detected. Recent studies have demonstrated that non‐cyanobacterial diazotrophs are widely distributed throughout the Arctic Ocean and can actively contribute to nitrogen fixation (Shiozaki et al. 2023; von Friesen, Farnelid, et al. 2025; von Friesen, Laber, et al. 2025). Understanding how these non‐cyanobacterial diazotrophs respond to ongoing environmental change will therefore be also critical for predicting future shifts in Arctic biogeochemical cycling.

In the Arctic Ocean, the reduction of sea ice has mitigated light limitation, such that nitrogen is the main factor limiting primary production (Tremblay et al. 2015). Consequently, understanding nitrogen dynamics is crucial for comprehending the Arctic ecosystem. The growing significance of nitrogen fixation in this region is poised to reshape our understanding of Arctic biogeochemical cycles. To improve the accuracy of future predictions for the Arctic marine ecosystem, precise modeling of the baseline processes is essential.

## Author Contributions

**Takuhei Shiozaki:** conceptualization, investigation, methodology, validation, funding acquisition, visualization, writing – original draft, writing – review and editing, project administration, formal analysis, software, data curation, supervision, resources. **Amane Fujiwara:** methodology, funding acquisition, investigation, validation, visualization, writing – review and editing, data curation, resources, software. **Eiji Watanabe:** investigation, software, resources, writing – review and editing, validation, methodology, data curation, funding acquisition. **Shigeto Nishino:** investigation, methodology, validation, writing – review and editing, resources, data curation, software, funding acquisition. **Naomi Harada:** resources, funding acquisition, project administration, writing – review and editing. **Akiko Makabe:** methodology, data curation, investigation, validation.

## Funding

This work was supported by Japan Society for the Promotion of Science (JP23H05411, JP24K22347, JP25K03239) and Ministry of Education, Culture, Sports, Science and Technology (ArCS II, ArCS III).

## Conflicts of Interest

The authors declare no conflicts of interest.

## Supporting information


**Dataset: S1.** Cruise data at each light depth in each year.

**Dataset: S2.** Surface PAR, nitracline depth, depth‐integrated biological activities, and contribution of N_2_ fixation to new production in each year.

**Table S1:** Pearson's correlation matrix among environmental parameters and biological productivity in each year.
**Table S2:** Expeditions, observation periods, and data sources for historical nutrient data.
**Figure S1:** (A–D) Spatial distributions of PAR, surface nitrate, surface chlorophyll *a*, nitrogen fixation, primary production, nitrate assimilation, nitrification, and contribution of nitrogen fixation to new production in each year. Solid lines indicate 100‐m isobaths.
**Figure S2:** Diazotroph community structure in surface water in each year. Black and orange numbers indicate stations in shelf and off‐shelf regions, respectively.
**Figure S3:** Relationships between (a) UCYN‐A2 abundance and nitrogen fixation and (b) temperature and UCYN‐A2 abundance in each year. Regression lines are plotted only for significant relationships (*p* < 0.05).
**Figure S4:** Timing of sea‐ice retreat in each year.
**Figure S5:** Differences in (A) primary production, (B) nitrate‐based new production, (C) nitracline depth, and (D) nitrogen fixation between shelf and off‐shelf regions. Lines on box plots indicate significant differences (*p* < 0.05, Wilcoxon rank sum test).

## Data Availability

The *nifH* sequence data generated for this study can be obtained from the DNA Data Bank of Japan Sequence Read Archive (BioProject Accession Number PRJDB5679 [https://ddbj.nig.ac.jp/search/entry/bioproject/PRJDB5679]). All other data are available in the Supporting Information Datasets S1 and S2.
