# Supplementary material for: Enhancing Role of Nitrogen Fixation in Biogeochemical Cycles of the Pacific Arctic
Source: Glob Chang Biol. 2026 May 15;32:e70910. doi: 10.1111/gcb.70910 (PMC13177275; doi:10.1111/gcb.70910)
Supplement: Supplementary file 3 — Table S1: Pearson's correlation matrix among environmental parameters and biological productivity in each year. Table S2: Expeditions, observation periods, and data sources for historical nutrient data. Figure S1: (A–D) Spatial distributions of PAR, surface nitrate, surface chlorophyll a, nitrogen fixation, primary production, nitrate assimilation, nitrification, and contribution of nitrogen fixation to new production in each year. Solid lines indicate 100‐m isobaths. Figure S2: Diazotroph community structure in surface water in each year. Black and orange numbers indicate stations in shelf and off‐shelf regions, respectively. Figure S3: Relationships between (a) UCYN‐A2 abundance and nitrogen fixation and (b) temperature and UCYN‐A2 abundance in each year. Regression lines are plotted only for significant relationships (p < 0.05). Figure S4: Timing of sea‐ice retreat in each year. Figure S5: Differences in (A) primary production, (B) nitrate‐based new production, (C) nitracline depth, and (D) nitrogen fixation between shelf and off‐shelf regions. Lines on box plots indicate significant differences (p < 0.05, Wilcoxon rank sum test). [file GCB-32-e70910-s002.docx]

Supplementary Information for

Enhancing role of nitrogen fixation in biogeochemical cycles of the Pacific Arctic

T. Shiozaki, A. Fujiwara, E. Watanabe, S. Nishino, N. Harada, A. Makabe

Correspondence to: shiozaki@g.ecc.u-tokyo.ac.jp

**This PDF file includes:**

Supplementary Methods

Table S1

Figs. S1 to S5

**Other Supplementary Materials for this manuscript include the following:**

Database S1 to S2

**Supplementary methods**

*Nitrogen fixation and primary production*

Samples were collected from depths corresponding to 100, 10, 1, and 0.1% of surface light intensity. Duplicate samples for incubation were collected in acid-cleaned 1.2-L or 2.3-L polycarbonate bottles. Samples for determining the initial ^15^N enrichment of particulate organic carbon were immediately filtered after collection. To prepare ^15^N_2_-enriched seawater, filtered surface seawater was passed through a Sterapore membrane unit (Mitsubishi Rayon Co., Ltd., Tokyo, Japan) at a flow rate of 500 ml min^-1^ for at least 10 min to completely remove dissolved N_2_. The degassed seawater was transferred into 1-L Tedlar bags, and ^15^N_2_ gas (99.8 atom%; SI Science, Kanagawa, Japan) was added at a ratio of 10 mL of ^15^N_2_ per 1 L of seawater. After complete dissolution of the ^15^N_2_, 50 mL of the ^15^N_2_-enriched seawater was added to each incubation bottle. Subsequently, ^13^C-labeled sodium bicarbonate (99 atom% ^13^C; Cambridge Isotope Laboratories, Inc., Andover, MA, USA) was added to achieve a final concentration of 200 µmol L^-1^, and the bottles were sealed with thermoplastic elastomer caps without any headspace. The volume of dissolved N₂ was calculated according to the equation of Weiss (1970). Although we assumed complete dissolution of the added ^15^N_2_, this calculation includes inherent uncertainty (White et al., 2020). It has been reported that ^15^N_2_ gas can be contaminated with ^15^N-labeled nitrate and ammonium during manufacturing (Dabundo et al., 2014). However, the ^15^N_2_ gas supplied by SI Science used in this study was confirmed to have negligible contamination of ^15^N species (Shiozaki et al., 2015). Samples were covered with a neutral-density screen to approximate in situ light levels and incubated in an on-deck incubator supplied with flowing surface seawater for 24 h. Incubations were terminated by gentle filtration onto pre-combusted (450°C for 6 h) GF/F filters (Whatman). Filters were stored at −20°C and transported to the shore-based laboratory. Subsequent treatments were conducted as described by Shiozaki et al. (2009). Nitrogen fixation and primary production rates were calculated using the equations given by Montoya et al. (1996) and Hama et al. (1983), respectively. To determine quantifiable nitrogen fixation rates, a sensitivity analysis was performed following Gradoville et al. (2017) and Montoya et al. (1996). Minimum quantifiable rates ranged from 0.05 to 3.30 nmol N L⁻¹ d⁻¹ in 2015; 0.04 to 1.42 nmol N L⁻¹ d⁻¹ in 2016; 0.11 to 28.56 nmol N L⁻¹ d⁻¹ in 2017; 0.03 to 0.99 nmol N L⁻¹ d⁻¹ in 2020 (Datasset S1). Depth-integrated rates were calculated from the surface to the 0.1% light depth using the trapezoidal integration method.

*Nitrate assimilation*

Samples for nitrate assimilation measurements were also collected from the 100%, 10%, 1%, and 0.1% light depths. For the surface and 10% light depths, samples were collected into four 1.2-L polycarbonate bottles, whereas for the other depths, two 1.2-L bottles were used. For the surface and 10% light depths, nitrate assimilation rates were determined using a Michaelis–Menten kinetics approach. ^15^N-labeled nitrate (99 atom% ^15^N; SI Science) was added to each bottle to achieve final concentrations of 10, 20, 100, and 2000 nM. For the 1% and 0.1% light depths, ^15^N-labeled nitrate was added to achieve final concentrations of 10–1000 nM. Samples were covered with a neutral-density screen to approximate *in situ* light levels and incubated for less than 3 h during daylight in the on-deck incubator described above. Incubations were terminated by gentle filtration onto pre-combusted GF/F filters. The analytical procedures for nitrate assimilation were identical to those described for nitrogen fixation.

For the Michaelis–Menten kinetics approach, when the *in situ* nitrate concentration (S) was ≤100 nmol L⁻¹, the uptake rates (ρₙ) obtained for each nitrate addition level (Sₙ) were fitted to Eq. 1 by least-squares regression to estimate the maximum uptake rate (ρₘₐₓ) and the half-saturation constant (Kₛ).

$\rho_{n}=\frac{\rho_{max}\times\left( S+S_{n} \right)}{K_{s}+\left( S+S_{n} \right)}$ (1)

The corrected nitrate assimilation rate (ρₖ) was then calculated from Eq. 2 using the estimated values of ρₘₐₓ and Kₛ.

$\rho_{k}=\frac{\rho_{max}\times S}{K_{s}+S}$ (2)

The enrichment of ^15^N-labeled nitrate in samples from the 1% and 0.1% light depths ranged from 0.4 to 84.6% (average 23.3±24.9%) in 2015; 1.9 to 92.9% (average 19.9±25.9%) in 2016; 2.0 to 76.6% (average 18.8±23.9%) in 2017; 1.2 to 47.6% (average 6.4±8.8%) in 2020. Because those except in 2020 exceeded the tracer-level threshold (<10%), nitrate assimilation rates at these depths may have been overestimated.

Daily nitrate assimilation rates were calculated based on the previously determined daytime-to-nighttime ratio of nitrate assimilation in the Chukchi Sea (Shiozaki et al., 2016). Depth-integrated rates were calculated from the surface to the 0.1% light depth using the trapezoidal integration method.

*Nitrification*

Samples for nitrification measurements were collected from the 100%, 10%, 1%, and 0.1% light depths, as for the other parameters. Duplicate samples were collected in acid-cleaned 0.3-L polycarbonate bottles. For determination of the initial ^15^N enrichment of nitrate + nitrite, samples were immediately filtered after collection through a 0.2-μm pore size cellulose acetate in-line filter (Dismic, Advantec MFS, Tokyo, Japan), and the filtrate was collected into 50-mL polypropylene bottles. For incubation experiments, ^15^N-labeled ammonium sulfate (99 atom% ^15^N; SI Science) was added to a final concentration of 99 nM. Samples were covered with neutral-density screens to adjust the light levels and incubated for 24 h in the on-deck incubator described above. After incubation, samples were filtered through a 0.2-μm pore size Dismic filter. Filtrates for isotope analysis were collected into 50-mL polypropylene bottles, and those for nitrate + nitrite concentration measurements were collected into 10-mL acrylic tubes. Nitrate + nitrite concentrations were determined on board using a QuAAtro 2-HR system (BL TEC K.K.). Filtrates for isotope measurements were frozen until analysis on land.

For isotope measurements, nitrate and nitrite in the filtrates were converted to N₂O by the denitrifier method (Sigman et al., 2001), as described previously (Kawagucci et al., 2018; Shiozaki et al., 2019). The δ^15^N of N_2_O was determined using a GasBench + PreCon trace gas concentration system interfaced to a Delta V Plus isotope ratio mass spectrometer (Thermo Finnigan, Waltham, MA, United States). Nitrate reference materials (IAEA-N3, USGS-32, and USGS-34) were used for calibration of δ^15^N to atmospheric N₂. Ammonia oxidation rates were calculated using the equation given by Raimbault et al. (1999).

When ambient nitrate + nitrite concentrations were ≤0.1 µM, isotope measurements could not be performed, and such samples were excluded from analysis. Depth-integrated rates were calculated from the surface to the 0.1% light depth using the trapezoidal integration method.

**Supplemental references**

Dabundo, R., Lehmann, M.F., Treibergs, L. et al. (2014). The contamination of commercial ^15^N_2_ gas stocks with ^15^N-labeled nitrate and ammonium and consequences for nitrogen fixation measurements. PLoS ONE 9(10), e110335, doi: 10.1371/journal.pone.0110335.

Hama, T., Miyazaki, T., Ogawa, Y. et al. (1983). Measurement of photosynthetic production of a marine phytoplankton population using a stable ^13^C isotope. Marine Biology 73, 31-36. doi: 10.1007/BF00396282.

Raimbault, P., Slawyk, G., Boudjellal, B. et al. (1999). Carbon and nitrogen uptake and export in the equatorial Pacific at 150°W: Evidence of an efficient regenerated production cycle. Journal of Geophysical Research 104, 3341-3356. doi: 10.1029/1998JC9000004.

Weiss, R.F. (1970). The solubility of nitrogen, oxygen, and argon in water and seawater. Deep-Sea Research 17, 721-735. Doi: 10.1016/0011-7471(70)90037-9.

White, A.E., Granger, J. Selden, C. et al. (2020). A critical review of the ^15^N_2_ tracer method to measure diazotrophic production in pelagic ecosystems. Limnology and Oceanography: Methods 18(4), 129-147. Doi: 10.1002/lom3.10353.

**Table S1** Pearson’s correlation matrix among environmental parameters and biological productivity in each year.

| **2015** | PAR | Temperature | Salinity | Nitrate | Ammonium | Phosphate | Chl *a* | N_2_ fix | PP | Nitrate assim | Nitrification |
| --- | --- | --- | --- | --- | --- | --- | --- | --- | --- | --- | --- |
| PAR | 1 |  |  |  |  |  |  |  |  |  |  |
| Temperature | 0.704^***^ | 1 |  |  |  |  |  |  |  |  |  |
| Salinity | 0.511^*^ | 0.826^***^ | 1 |  |  |  |  |  |  |  |  |
| Nitrate | -0.056 | 0.306 | 0.503^*^ | 1 |  |  |  |  |  |  |  |
| Ammonium | 0.013 | 0.476^*^ | 0.603^**^ | 0.941^***^ | 1 |  |  |  |  |  |  |
| Phosphate | 0.012 | 0.409 | 0.595^**^ | 0.984^***^ | 0.977^***^ | 1 |  |  |  |  |  |
| Chl *a* | -0.639^***^ | 0.809^***^ | 0.691^***^ | 0.056 | 0.232 | 0.143 | 1 |  |  |  |  |
| N_2_ fix | -0.337 | -0.330 | -0.223 | 0.236 | 0.113 | 0.172 | -0.258 | 1 |  |  |  |
| PP | 0.577^***^ | 0.722^***^ | 0.558^**^ | 0.199 | 0.293 | 0.227 | 0.885^***^ | -0.061 | 1 |  |  |
| Nitrate assim | 0.353 | 0.499^*^ | 0.584^**^ | 0.128 | 0.233 | 0.163 | 0.789^***^ | -0.248 | 0.741^***^ | 1 |  |
| Nitrification | -0.582^***^ | -0.214 | -0.008 | 0.472^*^ | 0.440^*^ | 0.433 | -0.216 | 0.395 | -0.140 | -0.048 | 1 |

| **2016** | PAR | Temperature | Salinity | Nitrate | Ammonium | Phosphate | Chl *a* | N_2_ fix | PP | Nitrate assim | Nitrification |
| --- | --- | --- | --- | --- | --- | --- | --- | --- | --- | --- | --- |
| PAR | 1 |  |  |  |  |  |  |  |  |  |  |
| Temperature | 0.538 | 1 |  |  |  |  |  |  |  |  |  |
| Salinity | 0.504 | 0.834^**^ | 1 |  |  |  |  |  |  |  |  |
| Nitrate | -0.792^**^ | -0.466 | -0.448 | 1 |  |  |  |  |  |  |  |
| Ammonium | -0.556^*^ | -0.376 | -0.644^*^ | 0.307 | 1 |  |  |  |  |  |  |
| Phosphate | -0.724^**^ | -0.437 | -0.636^*^ | 0.588^*^ | 0.509 | 1 |  |  |  |  |  |
| Chl *a* | 0.059 | 0.498 | 0.580^*^ | -0.075 | -0.222 | -0.025 | 1 |  |  |  |  |
| N_2_ fix | 0.367 | 0.502 | 0.437 | -0.484 | -0.459 | -0.329 | -0.185 | 1 |  |  |  |
| PP | 0.521 | 0.598^*^ | 0.659^*^ | -0.324 | -0.479 | -0.439 | 0.759^**^ | -0.045 | 1 |  |  |
| Nitrate assim | 0.415 | 0.498 | 0.514 | -0.175 | -0.434 | -0.300 | 0.706^**^ | -0.072 | 0.963^**^ | 1 |  |
| Nitrification | -0.313 | -0.410 | -0.545 | 0.182 | 0.280 | 0.475 | -0.325 | 0.189 | -0.404 | -0.201 | 1 |

| **2017** | PAR | Temperature | Salinity | Nitrate | Ammonium | Phosphate | Chl *a* | N_2_ fix | PP | Nitrate assim | Nitrification |
| --- | --- | --- | --- | --- | --- | --- | --- | --- | --- | --- | --- |
| PAR | 1 |  |  |  |  |  |  |  |  |  |  |
| Temperature | 0.504 | 1 |  |  |  |  |  |  |  |  |  |
| Salinity | 0.684^*^ | 0.685^*^ | 1 |  |  |  |  |  |  |  |  |
| Nitrate | 0.752^*^ | 0.324 | 0.551 | 1 |  |  |  |  |  |  |  |
| Ammonium | 0.584^*^ | 0.254 | 0.478 | 0.961^**^ | 1 |  |  |  |  |  |  |
| Phosphate | 0.726^*^ | 0.178 | 0.477 | 0.980^**^ | 0.944^**^ | 1 |  |  |  |  |  |
| Chl *a* | 0.838^**^ | 0.475 | 0.537 | 0.468 | 0.214 | 0.420 | 1 |  |  |  |  |
| N_2_ fix | -0.089 | 0.033 | 0.042 | -0.314 | -0.261 | -0.357 | -0.153 | 1 |  |  |  |
| PP | 0.857^**^ | 0.490 | 0.571 | 0.492 | 0.241 | 0.445 | 0.998^**^ | -0.149 | 1 |  |  |
| Nitrate assim | 0.777^*^ | 0.405 | 0.462 | 0.385 | 0.118 | 0.349 | 0.988^**^ | -0.180 | 0.985^**^ | 1 |  |
| Nitrification | -0.409 | -0.742 | -0.554 | -0.287 | -0.242 | -0.166 | -0.335 | 0.121 | -0.334 | -0.272 | 1 |

| **2020** | PAR | Temperature | Salinity | Nitrate | Ammonium | Phosphate | Chl *a* | N_2_ fix | PP | Nitrate assim | Nitrification |
| --- | --- | --- | --- | --- | --- | --- | --- | --- | --- | --- | --- |
| PAR | 1 |  |  |  |  |  |  |  |  |  |  |
| Temperature | 0.549 | 1 |  |  |  |  |  |  |  |  |  |
| Salinity | 0.192 | 0.757^*^ | 1 |  |  |  |  |  |  |  |  |
| Nitrate | 0.153 | 0.339 | 0.518 | 1 |  |  |  |  |  |  |  |
| Ammonium | 0.124 | 0.564^*^ | 0.706^*^ | 0.891^**^ | 1 |  |  |  |  |  |  |
| Phosphate | 0.196 | 0.517 | 0.693^*^ | 0.950^**^ | 0.974^**^ | 1 |  |  |  |  |  |
| Chl *a* | 0.807^**^ | 0.541 | 0.106 | -0.111 | -0.142 | -0.075 | 1 |  |  |  |  |
| N_2_ fix | n.a. | n.a. | n.a | n.a. | n.a. | n.a. | n.a. | 1 |  |  |  |
| PP | 0.703^*^ | 0.604^*^ | 0.165 | -0.101 | -0.145 | -0.087 | 0.933^**^ | n.a. | 1 |  |  |
| Nitrate assim | -0.257 | -0.143 | -0.021 | 0.467 | 0.177 | 0.260 | -0.126 | n.a. | 0.003 | 1 |  |
| Nitrification | -0.113 | -0.044 | 0.095 | 0.807^**^ | 0.526 | 0.612^*^ | -0.174 | n.a. | -0.122 | 0.851^**^ | 1 |

PAR, temperature, salinity, nitrate, ammonium, phosphate, and chlorophyll *a* (Chl *a*) were derived from surface data. Nitrogen fixation (N_2_ fix), primary production (PP), nitrate assimilation (Nitrate assim), and nitrification were derived from depth-integrated data.

^*^*p* < 0.05, ^**^*p* < 0.001

Table S2. Expeditions, observation periods, and data sources for historical nutrient data.

| Expedition | Observation period | Data source |
| --- | --- | --- |
| *Mirai* 2002 | September 2 – October 10, 2002 | http://www.godac.jamstec.go.jp/darwin/e |
| 2004 | September 1 – October 12, 2004 |  |
| 2008 | August 26 – October 9, 2008 |  |
| 2009 | September 7 – October 15, 2009 |  |
| 2010 | September 2 – October 16, 2010 |  |
| 2012 | September 3 – October 17, 2012 |  |
| 2013 | August 28 – October 21, 2013 |  |
| 2014 | August 31 – October 10, 2014 |  |
| 2015 | August 24 – October 22, 2015 |  |
| 2016 | August 22 – October 5, 2016 |  |
| 2017 | August 23 – October 1, 2017 |  |
| 2018 | October 24 – December 7, 2018 |  |
| 2019 | September 28 – November 10, 2019 |  |
| 2020 | September 19 – November 2, 2020 |  |
| *Araon* 2020 | August 4 – August 31, 2020 | https://kpdc.kopri.re.kr/search/694ee19e-1c0b-4a44-8fd6-e83c94992731 |
| BGEP 2003 | August 7 – September 7, 2003 | https://www2.whoi.edu/site/beaufortgyre/data/ctd-and-geochemistry/ |
| 2004 | July 29 – September 2, 2004 |  |
| 2005 | July 29 – September 1, 2005 |  |
| 2006 | August 5–- September 14, 2006 |  |
| 2007 | July 26 – August 31, 2007 |  |
| 2008 | July 17 – August 21, 2008 |  |
| 2009 | September 17 – October 15, 2009 |  |
| 2010 | September 15 – October 15, 2010 |  |
| 2011 | July 21 – August 18, 2011 |  |
| 2012 | August 2 – September 8, 2012 |  |
| 2013 | August 1 – September 2, 2013 |  |
| 2014 | September 21 – October 17, 2014 |  |
| 2015 | September 20 – October 16, 2015 |  |
| 2016 | September 22 – October 18, 2016 |  |
| 2017 | September 7 – October 2, 2017 |  |
| 2018 | September 7 – October 2, 2018 |  |
| 2019 | September 12 – October 4, 2019 |  |
| 2020 | September 14 – October 2, 2020 |  |
| CBL 2002 | August 19 – September 23, 2002 | http://psc.apl.washington.edu/HLD/CBL/CBL.html |
| ISSS 2008 | August 15 – September 26, 2008 | https://cchdo.ucsd.edu/cruise/90JS20080815 |

Expeditions conducted by the R/V *Mirai* (Japan) and R/V *Araon* (Korea) are labeled *Mirai* and *Araon*, respectively, with their expedition years. The Canada/USA Beaufort Gyre Exploration Project using the Canadian Coast Guard Ship Louis S. St-Laurent is labeled BGEP. The Chukchi Borderland Project and International Siberian Shelf Study conducted by the US Coast Guard Cutter Polar Star (USA) in 2002 and Yacob Smirniskyi (Russia) in 2008, respectively, are labeled CBL 2002 and ISSS 2008, respectively.

Fig. S1.

(A–D) Spatial distributions of PAR, surface nitrate, surface chlorophyll *a*, nitrogen fixation, primary production, nitrate assimilation, nitrification, and contribution of nitrogen fixation to new production in each year. Solid lines indicate 100-m isobaths.

Fig. S2.

Diazotroph community structure in surface water in each year. Black and orange numbers indicate stations in shelf and off-shelf regions, respectively.

Fig. S3.

Relationships between (a) UCYN-A2 abundance and nitrogen fixation and (b) temperature and UCYN-A2 abundance in each year. Regression lines are plotted only for significant relationships (*P* < 0.05).

Fig. S4.

Timing of sea-ice retreat in each year.

Fig. S5.

Differences in (A) primary production, (B) nitrate-based new production, (C) nitracline depth, and (D) nitrogen fixation between shelf and off-shelf regions. Lines on box plots indicate significant differences (*P* < 0.05, Wilcoxon rank sum test).

Dataset S1. (separate file)

Cruise data at each light depth in each year.

Dataset S2. (separate file)

Surface PAR, nitracline depth, depth-integrated biological activities, and contribution of N_2_ fixation to new production in each year.
